# No Effect of Weight on Judgments of Importance in the Moral Domain and Evidence of Publication Bias from a Meta-Analysis

**DOI:** 10.1371/journal.pone.0134808

**Published:** 2015-08-04

**Authors:** André L. A. Rabelo, Victor N. Keller, Ronaldo Pilati, Jelte M. Wicherts

**Affiliations:** 1 Department of Work and Social Psychology, University of Brasília, Brasília, Brazil; 2 Department of Methodology and Statistics, Tilburg University, Tilburg, The Netherlands; University of Cambridge, UNITED KINGDOM

## Abstract

In different cultures, people use the concept of weight to refer to important matters. Recent studies in grounded cognition suggested that experiences of weight affect unrelated judgments of importance in metaphor-congruent ways. Theories in grounded cognition and prime-to-behavior effects state that sensations of weight activate concepts of importance, which may affect morality-related variables that are influenced by judgments of importance. The present research aimed to test the effect of carrying a heavy (or light) clipboard on the perceived importance of helping and on the judged severity of moral transgressions. After finding no significant effects in two experiments, a third study explored whether these results were due to a specific lack of effect of weight on morality-related variables or to the concept of importance not being grounded in sensations of weight in Brazilian samples. Specifically, in Study 3 we attempted to replicate two seminal studies but found no significant effects. Together with evidence of publication bias in a meta-analysis of published studies, the current results suggest that the concept of importance may not be as universally grounded in sensations of weight as previously assumed. We discuss the implications of these results for grounded cognition theories and methodological and statistical aspects of priming studies.

## Introduction

People in different cultures use metaphors related to the concept of weight to talk about the importance, severity, gravity, or seriousness of objects, people, or situations. The concept of weight seems to be strongly tied to the concept of importance and its semantically associated concepts. Some experiments in the field of embodied cognition have found that even an incidental experience of weight (e.g., holding a heavy clipboard) may bias people toward judging and perceiving things as more important [1,2]

Theories of conceptual-metaphors offer explanations for these effects. They propose that certain concepts, usually ones that are abstract and harder to comprehend, map onto superficially dissimilar concepts, which are easier to grasp and are often concrete [3]. Thus, importance, an abstract concept, would be mapped onto the concrete concept of weight, and information processing regarding the latter would influence judgments regarding the former. For instance, Zhang and Li [4] found evidence coherent with this conceptual mapping by showing that the effect of weight sensations on importance judgments is mediated by the semantic activation of weight-related concepts.

Embodied cognition theories also offer explanations for the weight effect [5,6] and have inspired previous studies [1]. These theories hold that high-level information processing is composed of low-level simulations of perceptual experiences. Abstract concepts, such as importance, would be processed as simulations of weight experiences. Manipulating the intensity of these simulations (i.e., holding a heavy or light clipboard) would influence the higher-level importance judgments. Which theory best explains the effect is beyond our present scope and does not directly change our expectations regarding the studies reported in this manuscript. As argued by Landau et al. [3], embodied cognition and conceptual-metaphor theories are not mutually exclusive and may account for different instances of the effects. Barsalou [7], places these theories under the umbrella of grounded cognition which “reflects the assumption that cognition is typically grounded in multiple ways, including simulation, situated action, and, on occasion, bodily states”; pp. 619. Therefore, when referring to both embodied and conceptual-metaphor theories, we will hereafter use the more general term *grounded cognition*. We acknowledge that other authors have made important distinctions between these approaches [8], but we think that these differences were not relevant to the present article because both of them led us to a similar pattern of thinking and expectations about our research questions.

While grounded cognition theories focus on the nature of mental representations, recent theories in the social priming literature have explored the mechanisms by which active mental representations influence behavior. Loersch and Payne's [9] situated inference model proposes that priming effects on behavior and judgment are the result of three steps: first, the concept is activated via associative processes; second, the activated concept is misattributed to one's spontaneous response toward the current situation; finally, the questions afforded by the immediate situation determine what kind of effect the misattributed prime will have on subsequent judgment or behavior. Thus, in the case of the weight-importance effect, haptic experiences of weight would prime importance-related concepts, which would be misattributed to some aspect of the current situation, and the question afforded by the current situation would guide judgment or behavior.

The fact that the activated concept of importance can be misattributed to a wide variety of people, objects or situations allows, in principle, weight sensations to affect many different judgments or behavioral outcomes. Weight sensations have indeed been shown to affect impression formation [10], decision-making processes related to government funding [10], monetary value judgments [1], how influential a book is perceived to be [11], the perceived importance of reading nutritional information [4], and even meta-cognitive judgments of learning [12]. That is, the weight-importance effect has already been observed in a variety of distinct psychological and behavioral phenomena.

Past studies have also tested the effect of weight sensations on morality-related outcomes. Kouchaki, Gino, and Jami [13], for example, demonstrated that carrying a heavy backpack induces feelings of guilt, a moral emotion (as in the expression: *the heavy burden of guilt*), and makes individuals less willing to cheat. Interestingly, carrying a backpack did not affect importance judgments. Kouchaki et al. argued that the simulations that ground the emotion of guilt are modality-specific: they are simulations of carrying a load on your back and not of carrying a load in your arms, which would be more relevant to the abstract concept of importance. Two other studies tested the effect of weight on morality-related variables, albeit indirectly. Jostmann et al.’s [1] second study showed that participants who held a heavy clipboard (vs. light clipboard) thought that it was more important that decision-making was fair. Finally, Ackerman et al.’s [10] second study showed that holding a heavy clipboard (vs. light clipboard) made participants allocate more government funding to social issues. Although they are not conclusive, these findings can be interpreted as evidence that holding a heavy weight activates importance concepts that bias judgments of what should or should not be done, or of what is right or wrong. Here, we aimed to test whether carrying a heavy clipboard increases the perceived importance of helping, prosocial responses, and the severity of moral judgments.

Our first experiment tested whether holding a heavy clipboard would make participants think that helping someone was more important, and whether this would increase prosocial responses. Similarly, we expected a misattribution of activated importance concepts in social situations such as those involving moral transgressions. Our second experiment tested whether holding a heavy clipboard would increase the perceived severity or importance of moral transgressions.

Additionally, the current research tests the generalizability of the weight-importance effect to samples from a country different from those typically used in previous studies (e.g., samples from the USA), but where the language (Portuguese) also features the weight-importance metaphor. Although studies on embodied cultural cognition have shown cultural variation in the embodiment of concepts [14], the weight-as-importance metaphor is present in Portuguese in the same way as it is in English and other languages (e.g., *este é um assunto pesado*: this is a heavy matter; *você deveria pesar essas coisas de uma maneira mais balanceada*: you should weight these matters in a more balanced way; *isso dará maior peso aos seus argumentos*: this will add weight to your arguments). Additionally, there is some evidence corroborating this conceptual mapping in other countries, such as in samples from the Netherlands [1] and Asian countries [4].

As a result of the findings from the first two studies, and considering the increasing emphasis on replication of findings in psychology [15–17], we performed a third experiment that sought to conceptually replicate the effects found in Ackerman et al.’s [10] first and second studies. The replication would suggest whether the effect does not generalize specifically to morality-related variables in a Brazilian sample or whether weight sensations do not activate importance concepts even when studying a variable previously explored. Given the strong theoretical claims that have been maintained in the field of embodied cognition [18] and the potential applications that such manipulations could have [19], it is important to evaluate the generalizability of at least a part of this evidence.

## Experiment 1

If people were biased to perceive and judge things as more important after having recently experienced weight, they could subsequently perceive prosocial situations as more important (Hypothesis 1). In addition, based on the attribution model by Weiner [20], if people perceive the helping situation as more important, they should feel more sympathy for the person in need of help (Hypothesis 2) and be more prone to help that person (Hypothesis 3).

### Method

After agreeing to participate in our study, fifty-six passersby on a Brazilian university campus received a clipboard with a piece of paper containing a description of a person in need of help [20]. The scenario depicted a situation in which a classmate asks a colleague to borrow their class notes due to his/her absence from classes the week before. The scenario was followed by measures of the perceived importance of helping his/her classmate (one item), attribution of guilt to the person (four items; α = .73), sympathy for the person (ten items; α = .88), willingness to help the person (three items; α = .83), and demographic information (e.g., age, gender) [21,22]. Participants were randomly assigned to one of two conditions: light clipboard (298g) and heavy clipboard (1,560g).

As previous studies did not discuss the relevance of the relative weights used to manipulate experiences of weight, we chose clipboard weights similar to those used by Ackerman et al. [10] (Experiment 1: light (340.2 g) and heavy (2,041.2 g); Experiment 2: light (453.6 g) and heavy (1,559.2 g); mean difference between clipboards: *M* = 1,403.4). Two experimenters with a similar general appearance (i.e. two young white men), and similar knowledge of the hypothesis of the study, collected the data. Both were trained to approach participants in a similar way, with a pre-defined script, and to avoid eye contact or conversation during the experiment by turning away from the participant while he or she read the sheet of paper. The data file and syntax used for the data analysis of Experiment 1 is available at the Open Science Framework platform (accessible via: https://osf.io/nxm69/).

#### Ethics statement

In all experiments, the experimenters delivered a standardized oral informed consent to decrease the duration of the procedure and increase the chance that participants would complete the procedure (the standardized oral informed consent was as follows: “Hi, excuse me, I am conducting a research project. Would you like to participate? It is a study about opinions on social matters, it is quick and you would only have to answer this questionnaire. Your responses are anonymous and you can stop your participation anytime you want.”). The project was not submitted to an approving institutional review board because committees in Brazil only evaluate projects in the realm of medical and pharmacological research, which is not appropriate to evaluate research projects in areas such as social psychology. Despite the lack of appropriate committees for evaluating this kind of research in Brazil, the study was planned so as to strictly follow the Guidelines for ethical conduct of behavioral projects involving human participants proposed by the American Psychological Association [23]. Verbal consent was not formally documented. Most of the participants readily accepted to participate and no participant stopped their participation during any of the three experiments. No personal or identifying information was collected. The procedure took about three minutes and was the same across all the following studies reported in this paper.

### Results and discussion

Table 1 shows the means and standard deviations of the following analysis. The relationships tested showed homogeneity of variances and no significant differences regarding gender of participants or identity of the experimenter running the study. In addition, none of the ANOVA effects reached statistical significance. Perceived importance of the helping situation, *F*(1, 54) = .37, *p* = .55, *d* = .16, 95% CI [-0.27, 0.51], how guilty they perceived the person in need of help to be, *F*(1, 54) = .68, *p* = .41, *d* = .22, 95% CI [-0.23, 0.56]; sympathy for the person, *F*(1, 54) = .41, *p* = .52, *d* = .17, 95% CI [-0.26, 0.52]; and prosocial intentions toward the person, *F*(1,54) = .13, *p* = .72, *d* = .09, 95% CI [-0.32, 0.46], did not significantly differ between conditions and all of the effect sizes were small. The confidence intervals reported here are related to the effect size estimates (Cohen’s d).

**Table 1:** Influence of weight on the dependent measures of Experiment 1.

Measure	Clipboard	
	Light (N = 29)	Heavy (N = 27)
Importance	5.38 (1.43)	5.59 (1.19)
Perception of guilt	5.24 (1.04)	5.00 (1.16)
Sympathy	5.39 (1.06)	5.22 (.92)
Prosocial intentions	5.67(1.28)	5.78 (1.00)

Standard deviations are in parenthesis.

As hypothesized, a statistically significant and positive correlation was observed between sympathy and perception of the importance of the prosocial situation (Hypothesis 2), *r* = .57, *p* < .001, as well as between sympathy and prosocial propensity (Hypothesis 3), *r* = .61, *p* < .001. Although the data are not conclusive in this regard, they suggest that the weight-to-importance effect may not generalize to prosocial situations.

The small sample is a limitation of this study, although this sample size is typical for research in this field [1,10]. Effect sizes in these previous studies centered on *d* = .6 [1,10], which would gave our first study a power of .60. Another limitation of our study is that our hypothetical scenarios described the interaction between two third parties and participants would not themselves be involved in the social interaction described. Although we could have observed a different pattern of results with alternative scenarios, we believe that the basic reasoning derived from the theories described earlier justifies the prediction that holding a heavier clipboard would systematically bias individual’s perception of situations as being more important, even if they were not directly involved in the described interaction. If the question afforded by the context have different effects depending on whether the question regards oneself or someone else is a research question that could be best addressed by future studies. The correlations corroborate the relationships predicted by Weiner’s model, which attests to the validity of our measures, although there is no evidence that weight experiences can influence any process described in the model. The current data suggest that incidental weight experiences do not (strongly) affect prosocial perception, feelings, and intention as one might expect from theories of grounded cognition and prime-to-behavior effects.

## Experiment 2

We designed Experiment 2 to test the effect of weight experiences on moral judgments following the same reasoning of Experiment 1: weight experiences would activate concepts of importance, which would be misattributed to the scenarios describing moral transgressions. In turn, this would bias participants’ moral judgments. There is evidence that moral judgments can be influenced by different perceptual cues [24]. For example, cues of disgust can lead to harsher moral judgments [25]. Similarly, the experience of weight could bias people’s perception of situations involving moral transgressions as being more important, heavier, and morally severe, which would lead them to make harsher moral judgments.

### Method

For Experiment 2 to be more conclusive, we almost doubled the sample size. A a priori power analysis considering an alpha value of .05, a power value of .8, and a Cohen’s *d* = .6 (the typical effect size observed in earlier studies [1,10]) in order to perform a test of equal means between two independent samples indicated a necessary sample of approximately 88 participants. This power analysis was performed by means of the *pwr* package [26] for the R Statistical Package [27]. After agreeing to participate, ninety-six passersby on a Brazilian university campus received a clipboard with a piece of paper containing the translated and adapted version of four moral vignettes commonly used in previous studies [24,25,28] and one moral vignette similar to the other four. This last one described a situation in which a person buying food knowingly keeps a large sum of money he/she mistakenly received as change (the former vignettes we used were the sex, dog, wallet, and trolley vignettes from Schnall et al. [24]). We used the largely adopted back-translation method and also the Translation and Adaptation Review Form [29] to adapt the vignettes to Portuguese. Two fluent speakers of English and Portuguese were involved in this procedure. Translator A translated the original vignettes to Portuguese, and then Translator B back translated them to English. The final back-translated version was compared to the original scenarios to look for inadequacies in the language of the translated version. Participants judged how morally wrong each situation was on a ten-point scale (1 = not at all wrong; 10 = extremely wrong). The mean of all the responses given to each vignette was used as an index of moral judgment (five vignettes; α = .47).

Considering that different political orientations are associated with different moral foundations [30], it is possible that weight sensations only bias the transgressions of the moral foundations endorsed by the individual. In fact, Ackerman et al. [10] found that political orientation was a marginally significant covariate of the effect of weight on decisions to fund social issues, and they found that conservative individuals preferred less funding for social issues. Therefore, we measured political orientation with a ten-point scale ranging from extremely right/left-wing to evaluate if it could be a significant covariate of the effect of weight on the dependent measures. Participants were randomly assigned to one of two experimental conditions: light clipboard (423g) and heavy clipboard (2,260g). Six experimenters were trained to interact with participants in the same way that experimenters were trained in Experiment 1. In other respects, the procedure was identical to that in Experiment 1. We used five versions of the same questionnaire each with five different orders of presentation of the moral vignettes to decrease the probability that an order effect could influence our results. In all of these versions, firstly participants read and answered to the moral vignettes, and then they informed their sex, age, and political orientation, always in this order. The data file and syntax used for the data analysis of Experiment 2 is available at the Open Science Framework platform (https://osf.io/nxm69/).

### Results and discussion

We performed a factorial ANOVA with clipboard condition, experimenter, and participant’s sex as the independent variables; and the mean moral judgment score as the dependent variable. None of these analyses yielded statistically significant effects and all of the effect sizes were small, except for the main effect for gender: women exhibited harsher moral judgments (*M* = 7.69, *SD* = 1.49) than men, and this difference was associated with a medium effect size (*M* = 6.63, *SD* = 1.62) (*F*(1, 69) = 7.98, *p* = .006, *d* = .68, 95% CI [0.17, 0.98]). We also performed an ANCOVA in order to observe if clipboard condition would influence moral judgment after statistically controlling for the effect of political orientation, but political orientation was not a significant covariate (*F*(1, 88) = .35, *p* = .55, *d* = .12, 95% CI [-0.29, 0.53]). Subsequently, we performed a one-way ANOVA considering only clipboard condition as the independent variable. Participants in the light condition (*N* = 49, *M* = 7.12, *SD* = 1.59) and the heavy condition (*N* = 47, *M* = 7.18, *SD* = 1.64) did not significantly differ in the severity of their moral judgments and this analysis was associated with a small effect size, *F*(1, 94) = .03, *p* = .86, *d* = .04, 95% CI [-0.36, 0.43]. Considering the low reliability of the aggregated index, we also analyzed the influence of weight on each vignette separately. ANOVAs considering the vignettes as separate dependent variables showed no significant differences between conditions and were associated with similarly small effect sizes (see Table 2 for means, standard deviations, and test statistics).

**Table 2:** Influence of weight on each moral vignette of Experiment 2.

Moral vignette	Clipboard		
	Light (N = 49)	Heavy (N = 47)	Test statistics
Sex between siblings	8.16 (2.58)	8.66 (2.30)	*F*(1, 94) = .99, *p* = .32, *d* = .20, 95% CI [-0.20, 0.61]
Lost wallet	8.14 (2.67)	7.66 (3.08)	*F*(1, 94) = .68, *p* = .41, *d* = .17, 95% CI [-0.23, 0.56]
Dog eaten	5.10 (3.86)	6.02 (3.50)	*F*(1, 94) = 1.47, *p* = .23, *d* = .25, 95% CI [-0.15, 0.64]
Wrong change	8.88 (1.39)	8.47 (2.47)	*F*(1, 94) = 1.01, *p* = .32, *d* = .21, 95% CI [-0.19, 0.60]
Running over	5.33 (2.90)	4.98 (2.72)	*F*(1, 94) = .36, *p* = .55, *d* = .12, 95% CI [-0.28, 0.52]

Standard deviations are in parenthesis.

The power in this experiment was .83, based on an anticipated effect size of *d* = .6. As in Experiment 1, we were unable to find significant effects of weight on a dependent variable that had not been tested in the literature—moral judgments. Our results so far are inconsistent with previous studies and theories of grounded cognition or prime-to-behavior effects, or hint at important moderators related to cultural specificity of these earlier findings. Experiment 3 addresses one possible explanation for this inconsistency.

## Experiment 3

Given the differences between our first two experiments and previous studies on the weight-to-importance effect, the inconsistent findings may be due to a variety of moderators. It is possible that (1) the effect of activated importance concepts on morality-related variables does not appear in Brazilian samples or (2) that the concept of importance is not grounded in perceptual simulations of weight in Brazilian samples, despite the fact that Brazilians use the metaphor. To address these two possibilities, our third experiment aimed to replicate two effects of the Ackerman et al. [10] weight studies.

In their first study, Ackerman et al. had 54 passersby evaluate a job candidate by reviewing his resume on either a light or heavy clipboard. Subsequently, participants rated the applicant on a variety of measures concerning the importance the applicant attached to getting the job. They found that perceived seriousness of the applicant’s interest in attaining the job and the overall candidate rating were significantly greater in the heavy clipboard condition. As they did not report any measure of effect size, we calculated the effect size and corresponding confidence interval associated with this result, which is considerably wide and close to zero (*d* = .54, 95% CI [0.002, 1.09]). In their second study, 43 passersby were asked whether particular public issues should receive more or less government funding. The issues were either idiosyncratic and less important (e.g., public bathroom regulation) or socially relevant and more important (e.g., air pollution standards). The results indicated that participants holding the heavy clipboard judged that the socially relevant issues should receive significantly more funding than did the participants that held the light clipboard. We also calculated the effect size and corresponding confidence interval associated with this analysis (*d* = .56, 95% CI [-0.04, 1.17]). The clipboard weight did not significantly affect the participants’ judgment for the idiosyncratic issues.

### Method

A power analysis using the same parameters as those reported in the power analysis performed for Experiment 2 indicated that a total sample of approximately 88 participants would be necessary. One hundred passersby on a Brazilian university campus received a clipboard with a questionnaire containing a brief description of a job candidate and measures of the importance of getting the job very similar to those used by Ackerman et al. [10]: how well-placed the applicant would be in relation to the other applicants (1 = “Among the best”, 7 = “Among the worst”), how important it would be for the applicant to get the job (1 = “Not important at all”, 7 = “Extremely important”), how the applicant’s relationship with other colleagues would be (1 = “Terrible”, 7 = “Excellent”), how his/her performance would be in case he/she was hired (1 = “Terrible”, 7 = “Excellent”), and finally the overall impression of the applicant (1 = “Very negative”, 7 = “Very positive”). On the same sheet of paper there were eight campus issues (four were considered *a priori* to be more important and the other four less important) for which participants decided on an eleven-point scale whether funding should decrease (-5), stay the same (0), or increase (+5). Finally, there were two scales: one for subjective cognitive effort demanded by the survey (1 = “Practically nothing”, 7 = “I had to think a lot”), and another for perceived relevance of the survey (1 = “Extremely irrelevant”, 7 = “Extremely relevant”). The former was used by Ackerman et al. [10] to rule out the possibility that the rating of relevance was due to a self-perception of cognitive effort. We also measured participants' gender, height, weight, and political orientation for exploratory purposes. Similarly to Experiment 2, political orientation could influence decision-making processes in the allocation of money to particular issues [10], and so could be an important covariate related to the weight effect on the decision to allocate financial resources. Therefore, we predicted that accounting for political orientation would increase our chances of detecting an effect of experimental condition on the dependent measures. Participants were randomly assigned to two conditions: light (423 g) and heavy (2,260g) clipboard. Six experimenters were trained in the same way described in Experiment 1 before they collected the data.

We adopted the same data analysis procedures and details used by Ackerman et al. (2010) and our evidence of reliability and validity of measures was similar to theirs (with the exception that these authors did not measure some variables that we did, such as BMI, but the details regarding statistical tests and techniques—described next—were the same). We applied a maximum likelihood factor analysis with varimax rotation to the job candidate ratings measure (KMO = .78), which indicated that items loaded over .44 onto one factor. We computed a single measure of job candidate rating by averaging the five items (*α* = .78). Regarding the public issues, a maximum likelihood factor analysis with varimax rotation (KMO = .79) indicated that all of the eight items loaded over .36 onto two factors. We computed two measures: one of important issues (*α* = .65) and one of less important issues (*α* = .66). The data file and syntax used for the data analysis of Experiment 3 is available at the Open Science Framework platform (https://osf.io/nxm69/).

### Results and discussion

We performed a MANCOVA with clipboard condition as the independent variable, political orientation and BMI (Body Mass Index) as covariates, and the job rating index (JR), the important issues index (IS) and the less important issues index (LIS) as the dependent variables. Political orientation (JR: *F*(1, 82) = .50, *p* = .48, *d* = .14, 95% CI [-0.25, 0.53]; IS: *F*(1, 82) = 1.32, *p* = .25, *d* = .23, 95% CI [-0.16, 0.62]); LIS: *F*(1, 82) = .37, *p* = .54, *d* = .12, 95% CI [-0.27, 0.51]) and BMI (JR: *F*(1, 82) = .22, *p* = .64, *d* = .09, 95% CI [-0.30, 0.48]; IS: *F*(1, 82) = .52, *p* = .47, *d* = .15, 95% CI [-0.25, 0.54]); LIS: *F*(1, 82) = .007, *p* = .93, *d* = .02, 95% CI [-0.37, 0.41]) were not significant covariates—all of the confidence intervals were wide and included zero; all of the effect sizes were low; and no p-value was statistically significant—and clipboard condition did not influence any of the dependent variables after statistically controlling for these covariates. Table 3 indicates the number of participants per condition, means, and standard deviations of the following analysis. After this we performed a series of one-way ANOVAs considering only clipboard condition as the independent variable and each ANOVA had only one of the dependent measures as the dependent variable (i.e. job rating, important issues, less important issues, cognitive effort, relevance). An ANOVA indicated that there were no statistically significant differences between conditions regarding the judged fit of the candidate for the job and a small effect size was associated with this analysis, *F*(1, 98) = 2.33, *p* = .13, *d* = .31, 95% CI [-0.09, 0.70].

**Table 3:** Influence of weight on the dependent measures of Experiment 3.

Measure	Clipboard	
	Light (N = 48)	Heavy (N = 52)
Job candidate rating	5.07 (.96)	4.77 (.95)
Important issues	3.38 (1.12)	3.31 (1.10)
Less important issues	2.37 (1.36)	2.69 (1.21)
Cognitive effort	3.67 (1.42)	3.40 (1.38)
Relevance of research	4.90 (1.13)	4.79 (1.36)

Standard deviations are in parenthesis.

By means of a within-subjects t-test, we found a statistically significant difference between conditions of a medium effect size regarding the type of issue: participants allocated more money to important issues (*M* = 3.34, *SD* = 1.10) compared to less important issues (*M* = 2.54, *SD* = 1.29) (*t*(99) = 6.62, *p* < .001, *r* = .55), which attests to the validity of our measures. Just as Ackerman et al., we tested whether the more important issues were actually considered more important a priori by means of a *t*-test considering only the responses of participants in the light condition, as they are considered the control condition in Ackerman et al.’s experiment. Participants in the light condition allocated considerably more money to important issues (*M* = 3.37, *SD* = 1.12) compared to less important issues (*M* = 2.37, *SD* = 1.36) (*t*(47) = 5.48, *p* < .001, *r* = .50), which also attests to the validity of our measures. The effect size associated with this analysis in Ackerman et al. (2010) was larger than ours (*r* = .88) (see supporting online material for Ackerman et al.). One possible reason for this difference seems to be that our less important issues were considered to be more deserving of funding than their idiosyncratic issues (*M* = -.02, *SD* = 1.28; Ackerman et al., while our important issues were considered to be similarly worthy of funding as their social issues (*M* = 3.49, *SD* = 1.52; Ackerman et al.). Given that Ackerman et al. found an effect of clipboard weight only on the social issues and not in the idiosyncratic issues, the absence of similarly unimportant issues as those of Ackerman et al. would not reduce our chances of finding evidence for an effect.

Participants in different conditions did not significantly differ in how much investment they judged important issues should receive (*F*(1, 98) = .08, *p* = .78, *d* = .06, 95% CI [-0.33, 0.45]), or how much less important issues should receive (*F*(1, 98) = 1.45, *p* = .23, *d* = .25, 95% CI [-0.15, 0.63]), and these two analysis were associated with small effect sizes. We also analyzed all the issues individually and none of them were significantly affected by the experimental manipulation. Ackerman et al. [10] also found a main effect of gender on the important issues (women invested more than men) which was qualified by an interaction with clipboard condition (men in the light condition invested less on social issues than men in the heavy condition, and women did not differ between conditions in their ratings). By performing a factorial ANOVA (gender and clipboard condition as independent variables) we found a main effect of gender on important issues associated with a medium effect size (*F*(1, 96) = 7.50, *p* = .007, *d* = .59, 95% CI [0.15, 0.96]). Women (*M* = 3.71, *SD* = .84) invested more than men on important issues (*M* = 3.10, *SD* = 1.19), but we did not find a significant interaction of gender with clipboard condition (*F*(1, 96) = .38, *p* = .54, *η*_*p*_^*2*^ = .004). Participants in the light and heavy conditions did not differ in ratings of how much cognitive effort they exerted to answer the questionnaire, *F*(1, 98) = 0.88, *p* = .35, *d* = .19, 95% CI [-0.20, 0.58], and in perceived relevance of the research, *F*(1, 98) = .18, *p* = .67, *d* = .09, 95% CI [-0.31, 0.48].

The main effect size in Ackerman et al.’s [10] first study was approximately *d* = .54 and approximately *d* = .56 in their second study, so the achieved power of our experiment would be of about .85 considering the effect size of their study 1 and .87 considering the effect size of their Study 2. Both of Ackerman et al.´s studies exhibited a power of less than .62 with *d* = .55. Also, the confidence intervals for all of the effect size estimates are very close to zero or include zero (job candidate rating: Ackerman et al. (*d* = .54, 95% CI [0.002, 1.09]), our Experiment 3 (*d* = .31, 95% CI [-0.09, 0.70]); important issues: Ackerman et al. (*d* = .56, 95% CI [-0.04, 1.17]), our Experiment 3 (*d* = .06, 95% CI [-0.33, 0.45]).

## General Discussion

Our first and second studies were an attempt to test if carrying a heavy load affects the perceived importance of helping and the judged severity of moral transgressions. Our third experiment attempted to conceptually replicate the effect of weight on impression formation and on funding decisions [10]. All three studies were conducted with samples of Brazilian students to additionally test the generalizability of the weight effect to a different culture as used in earlier studies. Inconsistently with previous research and theory, we did not find statistically significant effects of weight on importance judgments and the effect sizes for the effects were much smaller than those previously reported.

There are some issues that might explain the discrepancy between our pattern of data and the one found in previous studies. First of all, Loersch and Payne [31] reviewed a number of possible variables that may interfere with the effect of primes on judgment and behavior. If the prime is blatant, for example, it is likely that it will not be misattributed to the target of focus, and no prime effect (or a contrasting prime effect) will be detected. We consider that this is unlikely to be the case. Past studies using similarly weighted clipboards did not report having this problem.

Another moderator mentioned by Loersch and Payne [31] is the distinctness of the target of focus. Targets of focus that are highly distinctive activate less ambiguous information, which reduces the probability of observing a priming effect. It is possible that the helping situation, the moral transgressions, the job candidate description, and the university issues were exceedingly distinctive and hence invulnerable to the weight-importance effect. This also seems unlikely given that all measures (except for the prosocial situation) were taken from previous priming studies that were able to show the predicted effects.

The most notable difference between our studies and past ones is the country in which they were done. Although further research could study specific processes, our results appear to suggest that the concept of importance is not strongly grounded in perceptual simulations of weight in Brazilian samples (or at least in samples from a university). This is surprising given that the initially proposed reasons for finding the effect were based on relatively universal features of development: people learn in their infancy that heavier objects require more physical strength and cognitive planning; they are more important than light ones, which leads to associations between weight and importance [1]. Given this reasoning, there is no apparent reason why one should not observe it in any given culture. In that sense, even considering that some embodiments might vary across cultures [14], it is justified to expect the observation of this effect in different cultures given the initial reasons pointed out by many authors studying this topic, although one could certainly expect some variation in effect sizes for any given psychological phenomena in a broad sense.

As described in the introduction of this article, this association was reflected in linguistic metaphors present in many countries. Despite weight-as-importance metaphors being present in Brazilian Portuguese, weight manipulations did not affect importance judgments. However, it is important to emphasize that the mere salience of a metaphor in a particular language is merely an indication that two concepts are mapped (embodied or grounded) on to each other. Just as Landau, Meyer, and Keefer [3] made clear, “insofar as metaphors operate at a conceptual, and not merely a linguistic, level, metaphoric transfer effects should obtain even in contexts where linguistic expressions of the relevant metaphors are not made salient” (pp. 1048). Thus, it is possible that there are unknown variables moderating the effect other than the presence or absence of the linguistic metaphor linking the concrete and abstract concepts, an issue that could be best addressed by future studies.

Finally, it is possible that previous experiments that found an effect of weight sensations on importance judgment are false positives [8], or at least subject to inflated outcomes due to publication bias and related biases. Certain aspects of scientific reporting and publishing increase the likelihood of false-positives and overestimated effect sizes in the literature [32–34]. The tendency for scientific journals to selectively publish positive results—called the “file drawer problem” [35]–could increase the amount of false positives [36]. Other aspects of scientific practice could increase the amount of false positives, such as the use of hidden degrees of freedom to attain significant results [33] and the habit adopted by many journals of demanding multiple studies even if they use underpowered samples [32]. Lakens [8] argued that these issues are possibly also problematic in the field of social embodiment, both at the empirical and the theoretical levels. The data used in studies of this line of research for theoretical inference is usually based on very small samples, which suggest a small evidentiary value for such studies, and the theoretical approaches are usually unsatisfactory in providing a framework for deriving empirically testable claims.

Francis, Tanzman, and Matthews [37] found evidence that the results reported by Ackerman et al. are probably “too good to be true”. Francis et al. applied the Test for Excess Significance (TES) to a set of articles published in the journal *Science* (including Ackerman et al.) and concluded that 83% of the articles analyzed are excessively successful in reaching statistical significance levels in their analysis, that is, they are too good to be true. TES estimates the probability of observing as many successful results (i.e. reaching statistical significance) as those actually reported assuming appropriate sampling, analysis, and reporting. Observing a low probability (*P*_*TES*_) from this test indicates that this assumption is questionable. From the eighteen studies considered in this analysis, Ackerman’s et al. results were amongst the five studies with the lowest values of probability—the studies with the most excessive successful results. As Francis et al. acknowledge, this does not mean that the theoretical claims made by the authors are necessarily wrong or that questionable actions were intentionally perpetrated, but it casts doubts on methodological aspects of the studies such as appropriate analysis and reporting. Francis et al. concluded that readers of these studies should be skeptical about their excessive success.

One result that is coherent with these statements is the conclusion that we reported in the results and discussion section of Experiment 3 by comparing the confidence intervals for the effect size estimates of Ackerman et al.’s studies and our results—that is, the fact that all of the confidence intervals were close to zero or included zero. TES has been criticized as a valid way to test if a study is too good to be true [38], although further descriptions of its problems is beyond the scope of this article. Yet, we were interested in seeing whether p-uniform [39] would also indicate publication bias in the literature on the effects of weight on importance. P-uniform is a new meta-analytic method that only considers statistically significant studies and should be able to estimate an underlying effect size that is not overly affected by publication bias. We ran the p-uniform method on the previously published studies that are reported in Table 4. As part of this method, we also ran a standard fixed-effect meta-analysis, which yielded a (biased) effect size estimate of *d* = .57 (*SE* = .052) 95% CI [0.47, 0.67]. However, this analysis also showed excessive homogeneity: Q = 4.70, DF = 24, p = .999993, which is an indication of publication bias. Indeed, the publication bias test with p-uniform showed clear publication bias; *L* = 5.1, *p* < .001. The bias corrected estimate given by p-uniform yielded a *negative* effect size estimate. Such a result is to be expected when many primary studies involved the use of practices in the collection and analyses of data that are aimed to obtain significance [39], such as choosing among different potential dependent variables or sequential testing [32,33]. To conclude, it is highly likely that the effects of weight on judgments of importance reported in the literature are subject to publication bias and that further research is needed to accurately estimate these effects in various contexts.

**Table 4:** Experiments of the effects of weight on importance perception and judgment.

Reference	N	Effect size (*SE*)	*p*
Ackerman et al. (2010) Study 1 [10]	54	.542 (.274)	.048
Ackerman et al. (2010) Study 2 [10]	43	.559 (.306)	.069
Chandler et al. (2012) Study 1 [11]	100	.419 (.201)	.037
Chandler et al. (2012) Study 2 [11]	60	.651 (.262)	.013
Chandler et al. (2012) Study 3 [11]	100	.474 (.201)	.019
Hafner (2013) Study 1 [40]	60	.520 (.259)	.046
Jostmann et al. (2009) Study 1 [1]	40	.696 (.320)	.031
Jostmann et al. (2009) Study 2 [4]	50	.597 (.282)	.035
Jostmann et al. (2009) Study 3 [4]	49	.616 (.288)	.033
Jostmann et al. (2009) Study 4 [4]	40	.711 (.320)	.027
Kouchaki et al. (2013) Study 1a [13]	30	.435 (.291)	.137
Kouchaki et al. (2013) Study 1c [13]	54	.586 (.274)	.033
Kouchaki et al. (2013) Study 2 [13]	51	.640 (.283)	.024
Kouchaki et al. (2013) Study 3 [13]	71	.541 (.239)	.024
Kouchaki et al. (2013) Study 4 [13]	124	.540 (.182)	.003
Maglio & Trope (2012) Study 2 [41]	71	.400 (.237)	.093
Zhang & Li (2012) Study 1 [4]	70	.563 (.241)	.020
Zhang & Li (2012)Study 2 [4]	78	.447(.227)	.050
Zhang & Li (2012) Study 4 [4]	80	.561(.226)	.013
Kaspar & Krull (2013)[42]	90	.637 (.214)	.003
Kaspar (2013) study 1 [43]	40	.013 (.331)	.002
Kaspar (2013) study 2 [43]	51	.636 (.283)	.025
Kaspar (2013) study 3 [43]	62	.571 (.256)	.026
Kaspar (2013) study 4 [43]	97	.414 (.204)	.043
Kaspar (2013) study 5 [43]	60	.625 (.261)	.017

We also emphasize that our experiments do not provide definitive evidence for the conclusion that experiences of weight do not affect importance judgments. Our evidence points to a lack of generalizability to samples from a country where such a metaphoric relationship between weight and importance exists and we believe that this is an important and unexpected finding for those interested in a precise understanding of this phenomenon and in boundary conditions for observing it. To obtain a stronger conclusion regarding this issue, international collaboration between independent laboratories, direct replications, and cross-cultural research are important steps. As the present authors are also interested in the theoretical understanding of how these bodily experiences might influence our social behavior and thought, as well as the understanding of confounds in psychological research and publication, we hope that these results will be seen as an invitation to improve our science collaboratively, and to more systematically understand what is really going on with the importance of weight experiences on social perception and judgment.
